# Deep learning and pathomics analyses predict prognosis of high-grade gliomas

**DOI:** 10.3389/fneur.2025.1614678

**Published:** 2025-08-11

**Authors:** Yuchen Zhu, Yuxi Gong, Weilin Xu, Xingjian Sun, Gefei Jiang, Lei Qiu, Kexin Shi, Mengxing Wu, Yinjiao Fei, Jinling Yuan, Jinyan Luo, Yurong Li, Yuandong Cao, Minhong Pan, Shu Zhou

**Affiliations:** ^1^Department of Radiation Oncology, The First Affiliated Hospital of Nanjing Medical University, Nanjing, China; ^2^The First School of Clinical Medicine, Nanjing Medical University, Nanjing, China; ^3^Second Affiliated Hospital, Zhejiang University School of Medicine, Hangzhou, China

**Keywords:** high-grade gliomas, deep learning, prognostic analysis, pathomics, IDH

## Abstract

**Objective:**

Utilizing pathomics to analyze high-grade gliomas and provide prognostic insights.

**Methods:**

Regions of Interest (ROIs) in tumor areas were identified in whole-slide images (WSI). Tumor patches underwent cropping, white space removal, and normalization. A deep learning model trained on these patches aggregated predictions for WSIs. Pathological features were extracted using Pearson correlation, univariate Cox regression, and LASSO-Cox regression. Three models were developed: a Pathomics-based model, a clinical model, and a combined model integrating both.

**Results:**

Pathological and Clinical Features were used to build two models, leading to a predictive model with a C-index of 0.847 (train) and 0.739 (test). High-risk patients had a median progression-free survival (PFS) of 10 months (p<0.001), while low-risk patients had not reached median PFS. Stratification by IDH status revealed significant PFS differences.

**Conclusion:**

The combined model effectively predicts high-grade glioma prognosis.

## 1 Introduction

According to a survey conducted by the Chinese Society of Oncology in 2022, the annual incidence rate of brain gliomas is approximately 6.4 per 100,000 individuals, establishing it as the leading primary malignant tumor in the central nervous system of adults (1). Of these, high-grade gliomas, classified as grades III–IV, make up the majority of malignant primary brain tumors in adults, representing about 62% (2). The mainstay treatment for high-grade gliomas involves a combination of maximal surgical resection and concurrent radiotherapy and chemotherapy utilizing temozolomide (3). Despite this, the 5-year overall survival rate for high-grade gliomas (grades 3 and 4) is still disappointingly low, between 6.6 and 30.9%, with a median survival time of 1.25 to 3 years. Moreover, emerging research has consistently demonstrated that patients experiencing disease progression within the first year exhibit a considerably poorer prognosis (4, 5). Consequently, there is an urgent imperative to actively identify prognostic markers prior to treatment initiation, as this could profoundly impact personalized clinical interventions and enhance patient survival rates.

Analyzing tissue slices histologically is crucial for diagnosing and planning tumor treatment, providing high-resolution images that reveal fundamental morphological characteristics. However, histological examination offers limited information, and the heterogeneity of biopsy materials, along with variations in pathology expertise, can affect final results. In this context, digital pathology can provide more objective diagnostic results by converting pathological images into digital format (whole-slide images; WSI) and acquiring extensive data, including quantitative aspects like morphology, texture, and biology (6, 7). This facilitates the assessment of pathological diagnoses and molecular expression levels. Additionally, deep learning has demonstrated remarkable results in interpreting medical images, being used for cancer detection, differential diagnosis, quantitative analysis of morphological phenotypes, and predicting patient survival. Satisfactory results have been achieved in many tumors (7–9).

The combination of histopathology and deep learning has been proven to be an accurate and practical method with predictive potential, widely used in identifying tumor types, distinguishing pathological grades, predicting treatment effects, and forecasting prognosis. It has been studied in various tumors, including bladder cancer, lung cancer and so on (8, 10). It is also widely used in gliomas (11, 12), however, there are limitations in the related research. Some studies focus on glioma patients with grades 2–4 (13), overlooking the significant heterogeneity present within these grades, which complicates the analysis of patient prognosis. Additionally, in clinical practice, high-grade gliomas exhibit greater invasiveness and malignancy, leading to poorer prognoses and shorter median survival times. Therefore, there is a greater clinical demand and value in studying high-grade gliomas therefore, studying high-grade gliomas is crucial. In addition, some studies have not compared multiple Deep Convolutional Neural Network Models to select the optimal one, which may affect the predictive results of the research (14, 15).

The main objective of this study is to establish a prognostic model for high-grade gliomas based on histopathology, which will evaluate patient prognosis and provide valuable insights to inform treatment decisions.

## 2 Method

### 2.1 Datasets and workflow

Between June 2016 and June 2023, we prospectively recruited patients diagnosed with high-grade gliomas confirmed by pathology at our center. For this retrospective analysis, inclusion criteria required patients to have: (1) no prior treatment before the confirmed diagnosis of glioma, (2) possessing postoperative histopathological findings and histological slides, and (3) relevant clinical information. Exclusion criteria included patients with: (1) Without histopathological reports and microscopic sections in the patient’s record, (2) with WSI of insufficient resolution for diagnostic use, and (3) lack of post-treatment follow-up data.

About 3 months after treatment, patients were closely monitored before undergoing MRI and functional magnetic resonance imaging (fMRI). The evaluation of recurrence followed the RANO criteria and was conducted by a multidisciplinary team (MDT) comprising experts from the Radiotherapy, Neurosurgery, and Radiology departments. The MDT performed a detailed assessment of clinical manifestations, the extent of enhancement, and the timing of recurrence for each individual patient. All patients underwent MRI scans, and the need for additional functional MRI methods like magnetic resonance spectroscopy (MRS) and perfusion-weighted imaging (PWI) was evaluated to aid in diagnosis. Disease progression was defined according to the following criteria: (1) Target lesions: An increase of at least 20% in the total of the longest diameters of CNS target lesions compared to the smallest total recorded during the study, along with at least one lesion exhibiting an absolute increase of 5 mm or greater, in addition to the required 20% relative increase. (2) Non-target lesions: Clear evidence of advancement in current enhancing non-target CNS lesions, the appearance of new lesions (except during immunotherapy), or definite progression of existing tumor-related non-enhancing (T2/FLAIR) CNS lesions (16). The primary outcomes of the study include progression-free survival (PFS), characterized as the time span during which patients display no indications of disease progression during or following treatment, in addition to the time from the initiation of treatment until the patient’s death.

**Figure 1:**
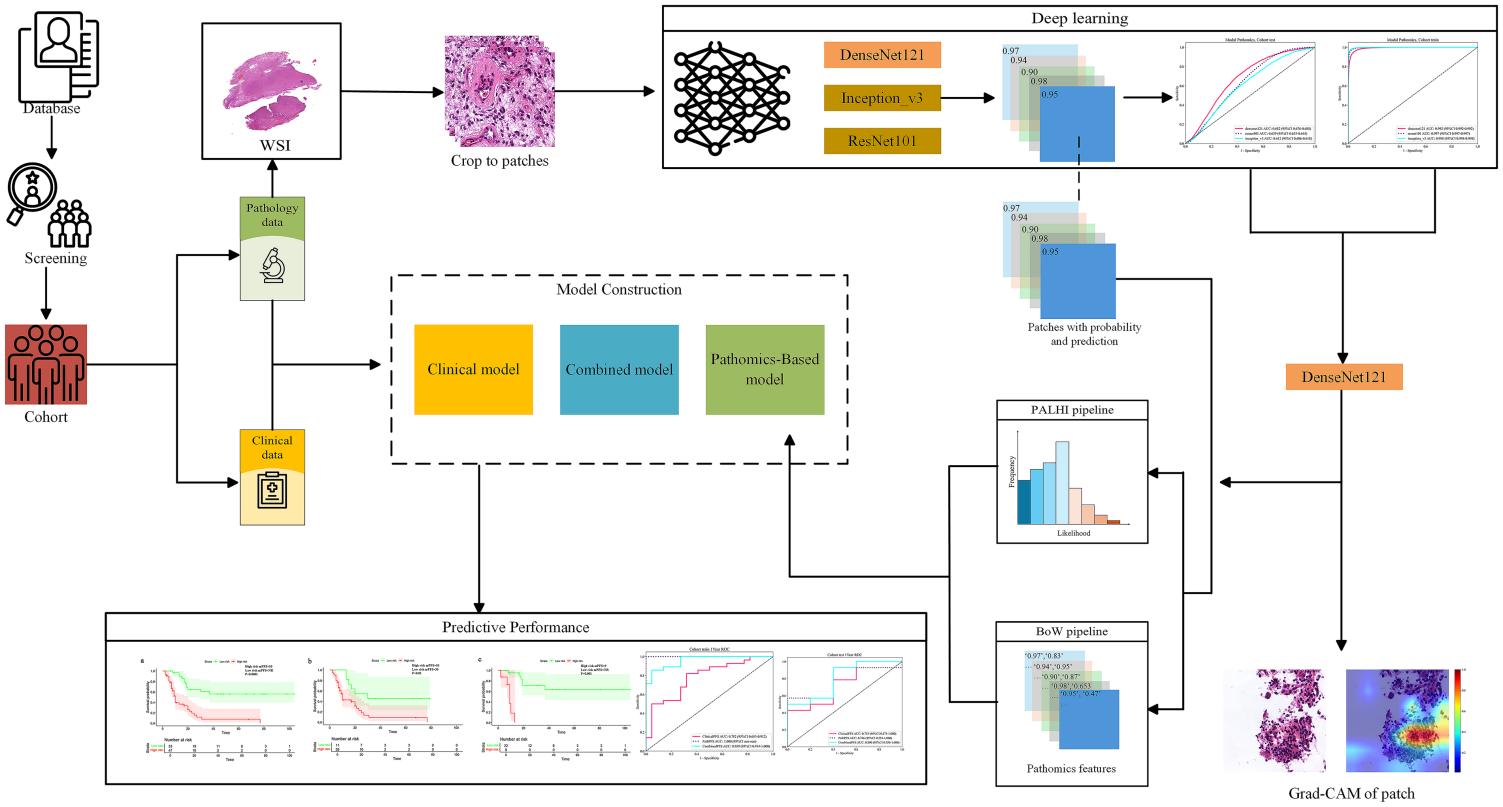
The workflow of the glioma pathological signature assessment method used in this study.

**Figure 2:**
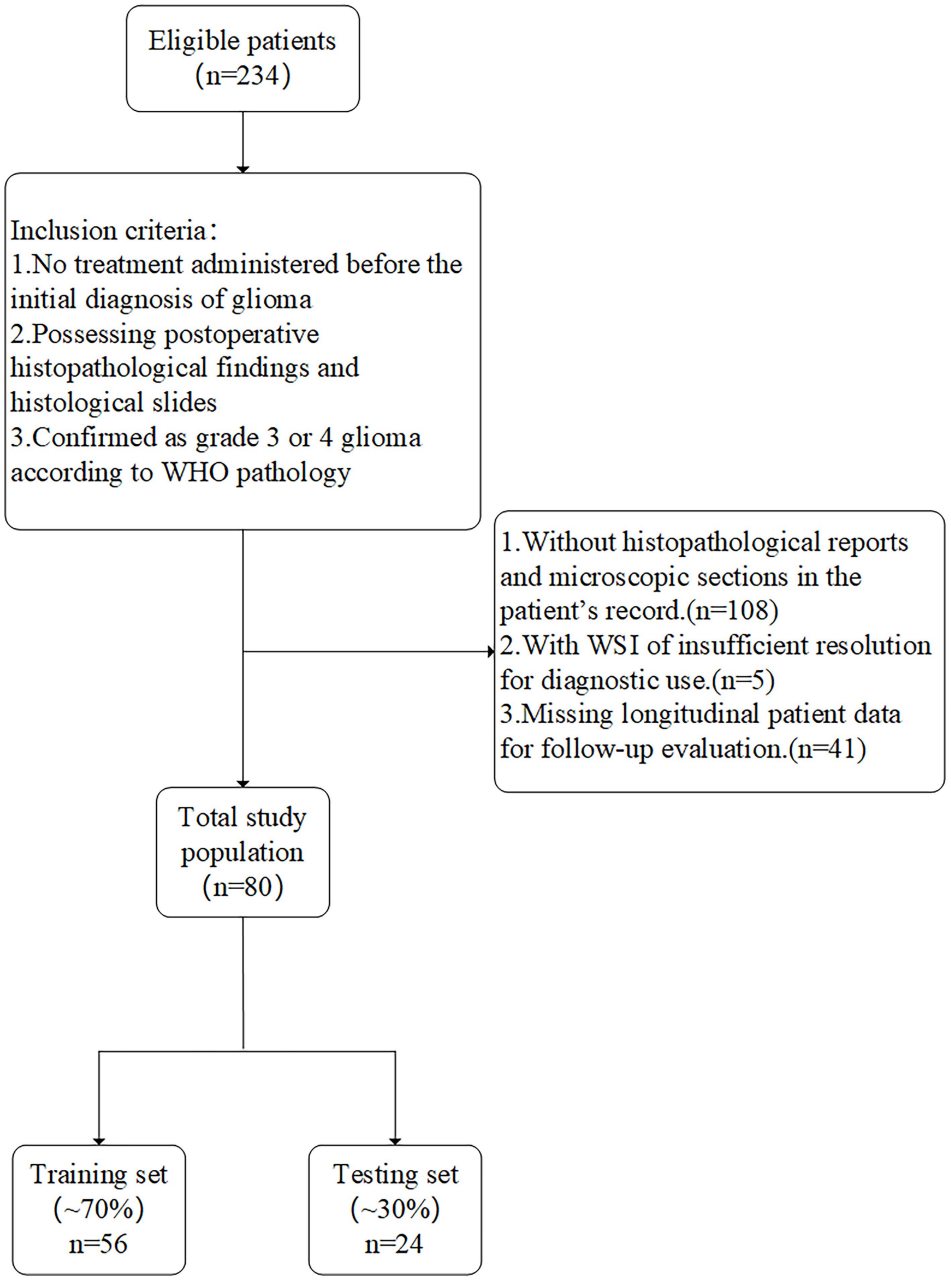
Flow chat of patient selection.

The analysis employed a retrospective cohort design to assess WSI data from a single institution, as shown in Figure 1. The initial phase involved image preprocessing techniques, including outlining the region of interest (ROI), cropping WSI into patches, removing white space, and normalization. Next, the cropped patches were trained using multiple architectures, and the trained deep models predicted labels for each patch, which were aggregated at the whole WSI level. Finally, pathological features were extracted through Pearson correlation analysis, univariate Cox regression, and LASSO-Cox regression analysis. The flowchart of patient selection and study framework is depicted in Figures 1, 2.

### 2.2 Treatment

Patients who met the eligibility criteria received a combination treatment approach. This involved radiotherapy delivered at a total dose of 60 Gy over a span of 6 weeks, divided into 30 fractions. Simultaneously, a daily dose of 75 mg/m^2^ of temozolomide was administered for 6 weeks, with a subsequent 4-week pause in treatment. Afterward, patients received maintenance therapy with temozolomide at a daily dose of 150–200 mg/m^2^ on days 1–5 of each 28-day cycle, up to a maximum of 6 cycles.

### 2.3 Clinical data acquisition

Before treatment, clinical characteristics were carefully collected from our center’s health information system (HIS). These comprehensive attributes encompassed demographic parameters such as age, sex, height, and weight, as well as clinical variables including a history of chronic diseases, family medical history, glioma grade, body mass index (BMI), pathological type of glioma, multifocality status, tumor distribution orientation, crossing of the midline, IDH status, and the presence or absence of necrosis. Additionally, the tumor’s measurements, including volume, were ascertained by precisely defining the region of interest (ROI) with ITKSNAP software.

### 2.4 Data processing

Our dataset comprises 80 WSI, and regions of interest (ROI) were delineated independently by two experienced pathologists using QuPath software. In cases where discrepancies existed between their annotations, these were resolved by a senior pathologist with 20 years of experience. Subsequently, we processed the digital whole-slide images (WSI) by segmenting them into 512 × 512-pixel tiles at 20× magnification for efficient management of their large size. During this process, we removed white backgrounds to eliminate tiles with sparse informative content, specifically those dominated by bright pixels. This selection resulted in over 12 million viable patches. All preprocessing tasks were conducted on the OnekeyAI Platform, using the OKT-crop_WSI2patch tool for cropping, OKT-patch2predict for background removal, and OKT-patch_normalize for color standardization. For more information, please consult Supplement A1.

### 2.5 Patch-level deep learning model training

Our deep learning pipeline features a dual-tier prediction framework that combines patch-level predictions with multi-instance learning to compile features from whole slide images (WSI). During training, we employed a weakly supervised learning approach, labeling patches based on the 1-year recurrence of the associated patient. We used the densenet121, inception_v3, and resnet101 architectures for training these patches. For a detailed description of the model structure and training parameters, please refer to Supplement A2.

### 2.6 Multi-instance learning for WSI fusion

Following the completion of our deep learning model’s training, we predicted labels and their corresponding probabilities for individual patches. The probabilities were subsequently merged using a classifier, resulting in predictions for the entire slide image (WSI). For more information, please consult Supplement A3.

### 2.7 Feature extraction & selection

In this study, we developed a pathological signature using a radiomics-like methodology, which combines patch-level predictions, probability histograms, and TF-IDF features. To remove redundant features, we applied Pearson’s correlation analysis (17), selecting those features with a correlation coefficient below 0.9. We further refined feature selection using univariate Cox regression and ranked the features by their *p*-values. The final feature set was determined through LASSO-Cox regression, where the optimal regularization parameter *λ* was selected via 10-fold cross-validation. Irrelevant features were then eliminated by setting their coefficients to zero. Additional details are in Supplement A4.

### 2.8 Model building

#### 2.8.1 Pathomics-based model

Following Lasso feature screening, Cox regression was employed to model the selected features and estimate the average expected survival time, resulting in the development of our pathological signature.

#### 2.8.2 Clinical model

We incorporated clinical characteristics into a Cox model, this modeling approach allowed us to predict the average expected survival time and ultimately create our clinical signature.

#### 2.8.3 Combined model

To validate the efficacy of a multi-omics approach, we merged the clinical signature and pathological signature using a Cox model, resulting in a combined model.

### 2.9 Model performance evaluation and survival analysis

Our study applied advanced analytical techniques to address challenges in medical image analysis. We utilized Cox proportional hazards models with L2 regularization for survival analysis and employed X-tile software to determine the optimal cut-off thresholds. This stratification enabled us to categorize patients into high-risk and low-risk groups, which were subsequently analyzed with Kaplan–Meier survival curves. The samples were stratified according to predicted hazard ratios (HRs), and a multivariate log-rank test was used to assess the importance of group separation. This comprehensive approach ensures a thorough evaluation of the predictive models’ effectiveness in clinical settings.

To evaluate the prognostic model, we use both micro and macro area under the curve (AUC) metrics, as well as the concordance index (C-index), to determine its effectiveness and select the best prognostic model based on their combined outcomes. In addition, we utilize the results of risk stratification combined with the patients’ molecular status to further refine the prognosis for patients.

### 2.10 Statistical analysis

The Shapiro–Wilk test was used to assess the normality of clinical characteristics. *t*-tests were applied to continuous variables that were normally distributed, and the Mann–Whitney *U* test was employed for those that were not. Statistical significance for categorical variables was determined using chi-square (*χ*^2^) tests. Detailed information on patient characteristics is available in Table 1. The machine learning model was developed and statistical analyses were performed using Python (version 3.7.12), Onekey (version 3.3.5), and scikit-learn (version 1.0.2), with the training process aided by an NVIDIA 4090 GPU, employing MONAI (version 0.8.1) and PyTorch (version 1.8.1) frameworks.

**Table 1:** Baseline clinical characteristics of patients.

Characteristics	The entire cohort number = 80	The train cohort number = 56	The test cohort number = 24	*p*-value
Age	55.12 ± 12.23	55.62 ± 12.34	53.96 ± 12.16	0.58
Height	165.30 ± 7.87	164.89 ± 8.21	166.25 ± 7.08	0.528
Weight	64.94 ± 9.73	64.24 ± 8.93	66.58 ± 11.42	0.327
BMI	23.74 ± 3.02	23.65 ± 3.00	23.95 ± 3.12	0.683
Tumor volume	46.30 ± 37.04	40.52 ± 29.63	59.77 ± 48.39	0.076
Tumor area	1.96 ± 1.05	1.93 ± 1.06	2.04 ± 1.04	0.58
Sex				1.0
Male	42 (52.50)	29 (51.79)	13 (54.17)	
Female	38 (47.50)	27 (48.21)	11 (45.83)	
Chronic				0.493
No	43 (53.75)	32 (57.14)	11 (45.83)	
Yes	37 (46.25)	24 (42.86)	13 (54.17)	
Family disease				1.0
No	78 (97.50)	55 (98.21)	23 (95.83)	
Yes	2 (2.50)	1 (1.79)	1 (4.17)	
Level				0.035
3	25 (31.25)	13 (23.21)	12 (50.00)	
4	55 (68.75)	43 (76.79)	12 (50.00)	
Pathological type				0.015
Glioblastoma	53 (66.25)	42 (75.00)	11 (45.83)	
Astrocytoma	10 (12.50)	5 (8.93)	5 (20.83)	
Oligodendroglioma	11 (13.75)	4 (7.14)	7 (29.17)	
Other	6 (7.50)	5 (8.93)	1 (4.17)	
Multifocal				0.122
No	72 (90.00)	48 (85.71)	24 (100.00)	
Yes	8 (10.00)	8 (14.29)	Null	
Tumor location				0.156
Left	35 (43.75)	21 (37.50)	14 (58.33)	
Right	42 (52.50)	32 (57.14)	10 (41.67)	
Multi-area	3 (3.75)	3 (5.36)	Null	
Beyond midline				0.367
No	49 (61.25)	32 (57.14)	17 (70.83)	
Yes	31 (38.75)	24 (42.86)	7 (29.17)	
IDH				0.45
No	50 (62.50)	37 (66.07)	13 (54.17)	
Yes	30 (37.50)	19 (33.93)	11 (45.83)	
Necrosis				0.054
No	26 (32.50)	14 (25.00)	12 (50.00)	
Yes	54 (67.50)	42 (75.00)	12 (50.00)	

## 3 Result

### 3.1 Patient characteristics

From June 2015 to June 2023, our center initially enrolled 234 patients with high-grade gliomas. After excluding 108 patients with missing histological slide, 41 patients with incomplete postoperative follow-up data, and five patients with blurry imaging data, the final analysis included a cohort of 80 patients (42 males and 38 females). Baseline characteristics such as age, sex, body mass index (BMI), pathological type, multifocality, and tumor volume were evaluated. Results of the between-group comparisons (*p* > 0.05) indicated that there were no significant differences between the two groups. The clinical data of the study are presented in Table 1.

### 3.2 Patch level efficiency

The AUC score analysis shows that DenseNet121 achieved the best test performance with an AUC of 0.682 (CI: 0.6761–0.6878). In comparison, ResNet101 and Inception V3 had AUCs of 0.639 and 0.612, respectively. DenseNet121 also demonstrated higher sensitivity (0.771) and a negative predictive value (NPV) of 0.882, though it had lower specificity (0.530) and a positive predictive value (PPV) of 0.336.

**Figure 3:**
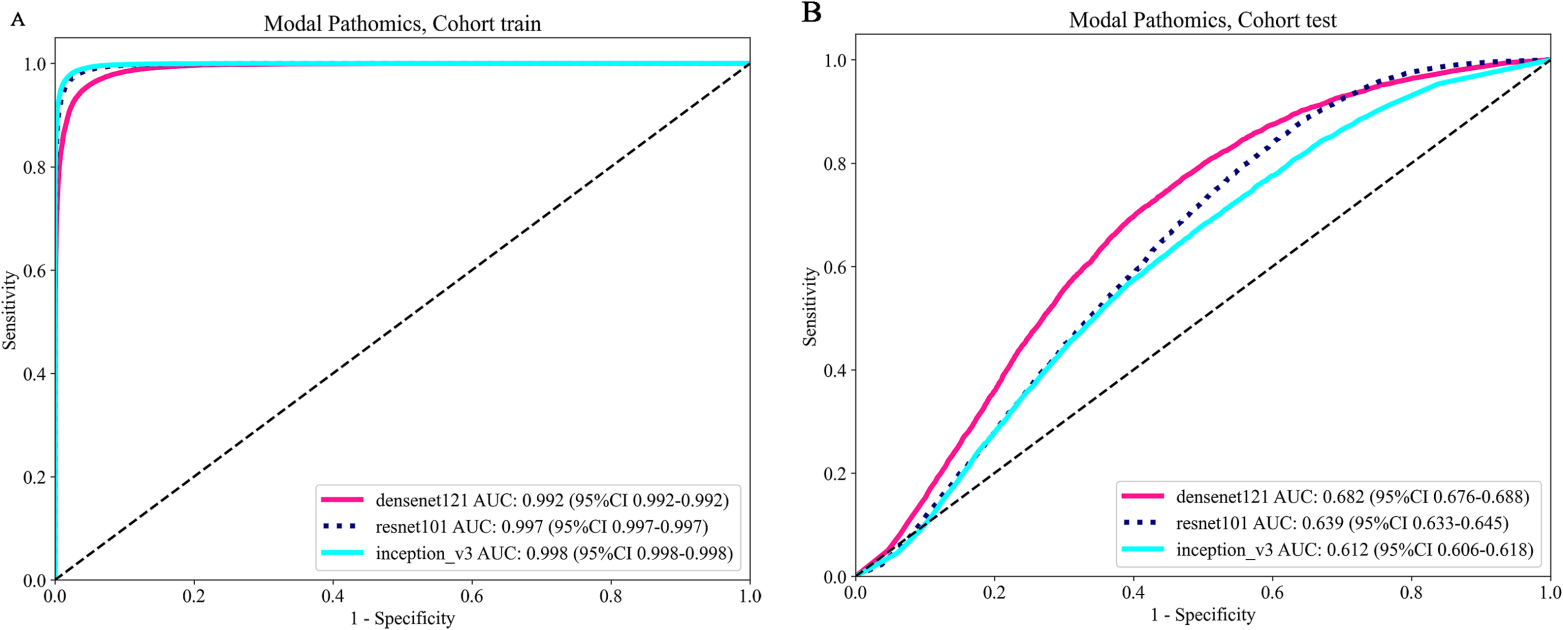
Showcases the ROC curves for each model’s performance on the train cohort **(A)** and the test cohort **(B)**.

Given its superior AUC and good balance between sensitivity and NPV, DenseNet121 is chosen for multiple instance learning in our study. This selection emphasizes the need for a predictive model that balances generalization and precision in real-world scenarios. Integrating DenseNet121 into our multi-instance learning framework is expected to enhance Pathological signature profiling. See Table 2 and Figure 3 for details.

**Table 2:** WSI level accuracy and AUC of each model.

Model name	Acc	AUC (95% CI)	Sensitivity	Specificity	PPV	NPV	Cohort
densenet121	0.954	0.992 (0.9915–0.9923)	0.952	0.957	0.954	0.955	Train
densenet121	0.586	0.682 (0.6761–0.6878)	0.771	0.530	0.336	0.882	Test
resnet101	0.974	0.997 (0.9968–0.9972)	0.974	0.974	0.972	0.975	Train
resnet101	0.502	0.639 (0.6332–0.6451)	0.843	0.397	0.302	0.891	Test
inception_v3	0.978	0.998 (0.9978–0.9981)	0.981	0.976	0.974	0.982	Train
inception_v3	0.542	0.612 (0.6057–0.6183)	0.681	0.499	0.296	0.835	Test

### 3.3 Grad-CAM visualization

We employed the gradient-weighted class activation mapping (Grad-CAM) method to visualize and assess the recognition capabilities of deep learning models on different samples, emphasizing the activations in the last convolutional layer that are pertinent to predicting cancer types. This helps in identifying image regions that significantly impact the model’s decision-making, offering insights into its interpretability. We also provide the prediction visualizations for some samples, and the related information can be found in Supplementary Figures 1, 2.

### 3.4 Model construction and predictive performance

#### 3.4.1 Model construction and signature comparison

In our study, pathomics-based, clinical, and combined models exhibited varying degrees of predictive accuracy. In the research related to PFS, the combined model had the highest C-index value in the training cohort, at 0.847, while the clinical model had the highest C-index value in the test cohort, at 0.746. This indicates that the integrated model achieved relatively stable performance after combining clinical information with pathological characteristics. For a comprehensive overview of the C-index values for each model, please refer to Table 3 in our publication.

**Table 3:** C-index in prediction PFS.

Model	Train cohort		Test cohort	
	C-index	*p*	C-index	*p*
Pathomics-based model	0.844	<0.05	0.710	<0.05
Clinical model	0.744	0.0006	0.746	0.0651
Combined model	0.847	<0.05	0.739	<0.05

#### 3.4.2 Time-dependent ROC analysis and development of a nomogram

**Figure 4:**
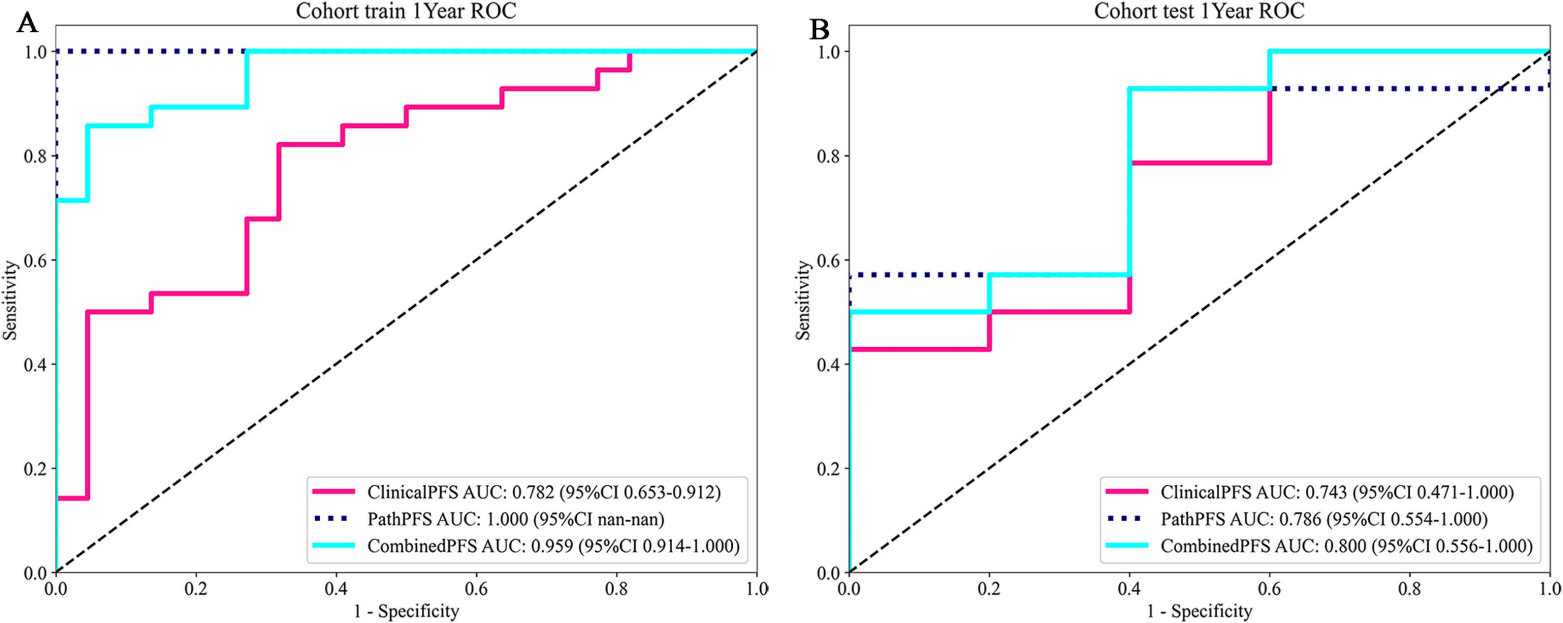
The ROC curves of deep learning algorithms for combined model in the train and test cohorts **(A,B)**.

In the training queue, the pathomics-based model achieved the highest AUC of 1.000, outperforming the clinical model (AUC = 0.782) and the combined model (AUC = 0.959). Within the test cohort, the combined model achieved the highest AUC of 0.800, with the pathomics-based model following at 0.786, and the clinical model at 0.743. These results indicate that the combined model exhibits superior AUC scores, particularly in the test queue, indicating its robust performance and potential predictive ability. This highlights its robustness and potential applicability in a clinical setting. The relevant ROC curve is shown in Figure 4.

**Figure 5:**
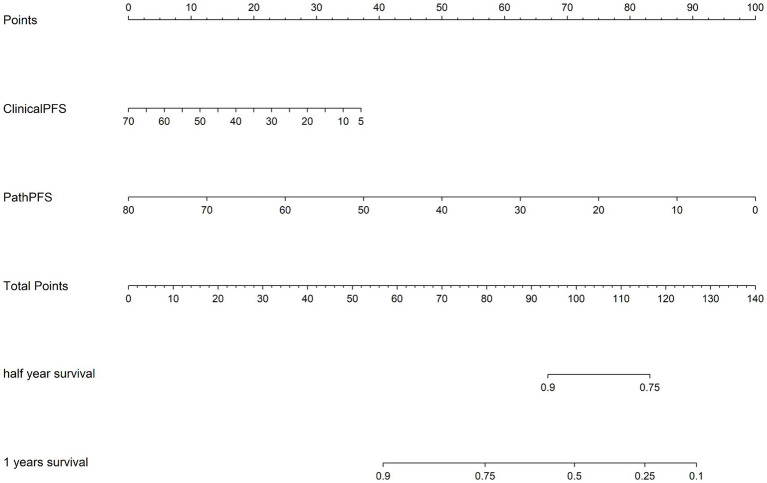
The corresponding nomogram showing the contribution of different factors. “ClinicalPFS” refers to the clinical features, “PathPFS” refers to the pathological features, “Points” refers to the numerical value assigned to each predictor variable based on its current value, and “Total points” refers to the sum of the points for all predictor variables.

In addition, we used time-dependent receiver operating characteristic (ROC) analysis to evaluate the predictive performance of the model, as detailed in Figure 5. “ClinicalPFS” refers to clinical features, and “PathPFS” refers to pathological features. “Points” refer to the numerical values assigned to each predictor based on its current value, while “Total points” is the sum of the points for all predictors, used to calculate the overall predictive result.

#### 3.4.3 Risk stratification and IDH status distinguish prognosis

In our research, the combined model outperformed other models in both the training and testing groups, establishing it as the best option for further analysis. Using this model, we stratified patients into high-risk and low-risk groups based on survival curves (*p* < 0.0001). The high-risk group had a median progression-free survival (PFS) of 10 months. In the IDH (−) population (*p* = 0.01), the high-risk group had a median PFS of 10 months, while the low-risk group had a significantly longer median PFS of 30 months. In the IDH (+) population (*p* = 0.001), the high-risk group had a median PFS of 9 months. These findings indicate a strong stratification effect, effectively distinguishing high-risk and low-risk patients. The relevant survival curve is shown in Supplementary Figure 3.

## 4 Discussion

In our study, the histopathology of WSI data was used to develop a predictive model for the prognosis of high-grade gliomas. The combined model demonstrated good prognostic value in both the training cohort (C-index = 0.847, AUC = 0.959) and the testing cohort (C-index = 0.739, AUC = 0.800), and was comprehensively evaluated as the optimal model. Furthermore, patient stratification based on the combined model, particularly focusing on the IDH status, improved survival prediction and provided additional information for prognostic stratification.

Pathomics seeks to investigate the microscopic patterns present in digital histopathology slides or whole slide images (WSI) using a high-throughput approach (18). The tumor microenvironment (TME) can be thoroughly characterized by “subvisual” quantification of the presence and spatial arrangement of different cell types, such as immune cells, fibroblasts, and blood vessels, thereby successfully predicting the prognosis of high-grade gliomas and providing options for treatment plans (18, 19). Compared to radiomics, which focuses on the macroscopic level, pathomics has a significant advantage in spatial resolution (20, 21). In addition, research by Dia et al. (20) demonstrated that pathomics may have stronger predictive capabilities even in the presence of significant exceptions.

Previous studies have used histopathology models to predict the prognosis of high-grade gliomas. However, most of these studies did not employ deep learning techniques. Recent research has shown that deep analysis models possess better generalization capabilities and interpretability (22, 23). The strong generalization capability allows the model to accurately classify and predict patient prognosis (23), while interpretability offers enhanced transparency, enhancing human understanding of internal workings and decision-making, while supporting bias correction (22).

Our study differs from previous articles in key aspects. Rathore et al. (24) included both low-grade and high-grade gliomas, introducing significant tumor heterogeneity that could impact the accuracy of prognosis prediction. He’s et al. (14) study did not conduct multi-model comparisons, as different models have their own advantages and disadvantages. By comparing the performance of different models, we can select the most suitable model to improve overall prediction accuracy and efficiency (25, 26). Additionally, Jiang’s et al. (15) deep learning study overlooked multiple instance learning, which can enhance model performance and improve the accuracy of outcome prediction, playing a crucial role in prognosis prediction (27, 28).

IDH status is an important molecular marker in gliomas. The IDH wild-type usually indicates a poor prognosis (29, 30). In recent years, an increasing number of studies have utilized pathomics to predict IDH status. In the study by Zhao et al. (31), pathological features were effectively used to predict patients’ IDH status, with the model’s AUC value exceeding 0.9. There are also studies that have combined IDH status and pathological features to construct prognostic prediction models for gliomas and analyze the relationship between them. In the study by Chunduru et al. (32), whole-slide images (WSIs) of low-grade and high-grade gliomas were collected. After feature extraction, they were integrated into risk score features (SDL risk score). Further research confirmed that this score has a strong correlation with IDH status in genetic subtypes, and the combination of both is equally effective in predicting patient survival. This study, based on pathomics, constructs a prediction model for the progression of high-grade gliomas. The model is also applied to groups with different IDH statuses for stratification and survival analysis. This model can identify high-risk progression populations within the IDH-mutated group, enabling clinicians to better manage patients and adjust treatment strategies.

In practical applications, we can utilize a combined model to stratify patients for risk assessment, thereby assisting the work of clinical practitioners. Firstly, clinicians can leverage the risk stratification information provided by the model to customize treatment plans, selecting appropriate therapeutic strategies based on the predicted outcomes. Our research findings indicate that the model is effective in identifying high-risk patients. For these high-risk patients, clinicians may consider the following measures in their treatment decision-making: (1) Whether to adjust the radiation dose and irradiation range during the concurrent radiotherapy combined with TMZ. (2) Whether to consider combining electric field therapy or anti-angiogenic therapy during the concurrent radiotherapy phase and the 6-cycle oral TMZ phase (33, 34). (3) Consider extending the duration of maintenance therapy to achieve a better prognosis. (4) Attempt to administer medication preoperatively to obtain better surgical conditions and improve the quality of life after treatment. (5) Conducting relevant clinical trials targeting this population. (6) For patients with a poorer prognosis, more frequent follow-ups can help detect changes in their condition earlier, allowing for timely treatment. For low-risk patients, the following measures can be considered: (1) Consider appropriately reducing the radiotherapy dose to lower the side effects experienced by the patient during radiotherapy. (2) Appropriate extension of patient follow-up intervals can reduce their economic burden. In future clinical practice, we hope to conduct non-inferiority clinical trials and other studies to further verify the feasibility of these measures. Additionally, by referencing the predictive outcomes from the model, clinicians can engage in more candid and informed discussions with patients, collaboratively exploring treatment options. Ultimately, the ability of the stratification model to accurately predict outcomes can help healthcare institutions better allocate resources, such as in radiotherapy planning and subsequent care management. By focusing on high-risk patients, healthcare providers can optimize scheduling and resource utilization, thereby enhancing overall healthcare efficiency.

This is a single-center study lacking external validation. Due to its interdisciplinary nature and issues related to the management of pathological slides, pathological slides for some patients could not be obtained. This ultimately limited the sample size included in the study, consequently affecting the generalization ability of the research model to some extent. Moreover, in the design of this study’s analysis, we only utilized IDH status for subgroup analysis and did not analyze other molecular markers, such as MGMT methylation and 1p/19q deletion status. Furthermore, we did not investigate the correlation between IDH status and pathological features, which resulted in a lack of analysis regarding the biological interpretability of pathomics. Finally, this study only predicted progression-free survival (PFS) and did not include the important overall survival (OS) indicator. In the future, we plan to expand the sample size and conduct multi-center collaborations, while incorporating more molecular features and further exploring their relationships with pathological features. Moreover, we will utilize the model to further predict patients’ survival outcomes to provide insights for selecting clinical treatment options.

Our study established a prediction model based on WSI to forecast the prognosis of high-grade gliomas. The combined model, integrating clinical data and pathological features, outperformed other models in terms of predictive performance. Furthermore, the model’s ability to classify patient survival based on the IDH status enhanced its predictive capacity. This study provides valuable insights for improving personalized treatment strategies and prognostic assessment of high-grade gliomas.

## Data Availability

The datasets presented in this article are not readily available because this dataset contains patient privacy. Requests to access the datasets should be directed to SZ, zhoushu164086035@126.com.
