# Supplementary material for: Deep learning and pathomics analyses predict prognosis of high-grade gliomas
Source: Front Neurol. 2025 Aug 11;16:1614678. doi: 10.3389/fneur.2025.1614678 (PMC12376429; doi:10.3389/fneur.2025.1614678)
Supplement: Supplementary file 1 [file Data_Sheet_1.docx]

Supplementary Materials

Deep Learning and Pathomics Analyses predict prognosis of high-grade gliomas

Contents:

Supplement A1. Image preprocessing parameters

Supplement A2. Slice Level Model Training

Supplement A3. Multi Instance Learning for WSI Fusion

Supplement A4. Feature Selection

Supplementary Figure 1. Gradient-weighted Class Activation Mapping

Supplementary Figure 2. Visualize predictions

Supplementary Figure 3. Risk stratification and IDH status distinguish

Prognosis

Supplement A1. Image preprocessing parameters

1. Image cropping

Image cropping is performed using the OpenSlide library, with a 20x magnification and patches of size 512*512.

1. Background removal

The removal of the white background is based on image brightness. Specifically, for a 3-channel RGB image, the brightness is calculated using the formula: sqrt(0.241 * (r ** 2) + 0.691 * (g ** 2) + 0.068 * (b ** 2)). Then, a threshold of 214 is used to remove images that are too "white."

1. Normalization

Color standardization is performed based on the Macenko method.

All the tools were developed by the R&D engineers on the OnekeyAI platform, who used the platform's tools, specifically OKT-crop_WSI2patch, OKT-patch2predict, and OKT-patch_normalize, to accomplish the tasks. The URL of the platform is:<https://github.com/OnekeyAI-Platform/onekey>

Supplement A2. Slice Level Model Training:

- Model Structure

CNN Characteristics: Convolutional Neural Networks (CNNs) are particularly effective for image processing due to their ability to capture hierarchical patterns and features through convolutional layers. These networks employ filters to perform convolution operations that extract spatial hierarchies of features, making them adept at handling image data.

Data Augmentation: To ensure a uniform input distribution, Z-score normalization was applied to the RGB channels of images. Our model benefited from online data augmentation techniques such as random cropping and flipping, both horizontal and vertical, to increase the variability and robustness of the training data. For testing, normalization was the sole preprocessing step to maintain consistency.

Training: The use of transfer learning, with initialization from pre-trained ImageNet weights, leverages rich feature representations developed for broad visual recognition tasks, thus enhancing the model’s adaptability to our specific medical imaging context. The adoption of a cosine decay learning rate algorithm further aids in managing the learning rate throughout training to avoid local minima and stabilize convergence. Specifically, this approach adjusts the learning rate according to the formula:

η_t=η_min^i+1/2 (η_max^i-η_min^i )(1+cos(T_cur/T_i π))

The minimum and maximum learning rates are set to 0 and 0.01, respectively, with a cycle of 10 epochs. Training was optimized using Stochastic Gradient Descent (SGD), and softmax cross-entropy was employed for calculating loss, which enhances the discriminative training of deep networks for multi-class classification.

- Training Parameters

The relevant parameters are: Batch_Size=32, epoch=10, lr=0.01. The method uses cosine-based decay, and the optimizer is SGD. We employed early stopping, stopping the training if the iteration count does not decrease after 32 iterations.

Supplement A3. Multi Instance Learning for WSI Fusion:

Following the completion of the training phase for our deep learning model, we advanced to the stage of predicting labels and their corresponding probabilities for each individual patch extracted from whole slide images (WSI). These probabilities were not evaluated in isolation; instead, they were integrated using sophisticated classifiers to derive cohesive predictions at the WSI level. To effectively aggregate the probabilities associated with individual patches, we implemented two innovative machine learning methodologies designed to capture and utilize the complex data structure inherent in WSIs:

1. Patch Prediction: Each slice was analyzed using the deep learning model to derive probabilities and labels, denoted as $\boldsymbol{Patc}\boldsymbol{h}_{\boldsymbol{prob}}$ and $\boldsymbol{Patc}\boldsymbol{h}_{\boldsymbol{pred}}$, retained to two decimal places.
2. Multi Instance Learning Feature Aggregation:
   - Histogram Feature Aggregation:
     - Distinct numbers were treated as "bins" to count occurrences across types.
     - Frequencies of $\boldsymbol{Patc}\boldsymbol{h}_{\boldsymbol{prob}}$ and $\boldsymbol{Patc}\boldsymbol{h}_{\boldsymbol{pred}}$ in each bin were tallied and normalized using min-max normalization, resulting in $\boldsymbol{Hist}\boldsymbol{o}_{\boldsymbol{prob}}$ and $\boldsymbol{Hist}\boldsymbol{o}_{\boldsymbol{pred}}$.
   - Bag of Words (BoW) Feature Aggregation:
     - A dictionary was constructed from unique elements in $\boldsymbol{Patc}\boldsymbol{h}_{\boldsymbol{prob}}$ and $\boldsymbol{Patc}\boldsymbol{h}_{\boldsymbol{pred}}$.
     - Each slice was represented as a vector noting the frequency of each dictionary element, with a TF-IDF transformation applied to emphasize informative features.
     - This resulted in a BoW feature representation for each slice, encapsulating both the presence and significance of features.
3. Feature Early Fusion: We integrated $\boldsymbol{Hist}\boldsymbol{o}_{\boldsymbol{prob}}$, $\boldsymbol{Hist}\boldsymbol{o}_{\boldsymbol{pred}}$, $\boldsymbol{Bo}\boldsymbol{w}_{\boldsymbol{prob}}$, and $\boldsymbol{Bo}\boldsymbol{w}_{\boldsymbol{pred}}$ using a feature concatenation method ($\boldsymbol{\oplus}$), combining these into a single comprehensive feature vector:

$$\boldsymbol{featur}\boldsymbol{e}_{\boldsymbol{fusion}}\mathbf{=}\boldsymbol{Hist}\boldsymbol{o}_{\boldsymbol{prob}}\boldsymbol{\oplus}\boldsymbol{Hist}\boldsymbol{o}_{\boldsymbol{pred}}\boldsymbol{\oplus}\boldsymbol{Bo}\boldsymbol{w}_{\boldsymbol{prob}}\boldsymbol{\oplus}\boldsymbol{Bo}\boldsymbol{w}_{\boldsymbol{pred}}$$

Supplement A4. Feature Selection

Feature Selection

Correlation Analysis: We employed Pearson's correlation coefficient to identify highly repeatable features. Features with a correlation coefficient exceeding 0.9 led to the retention of only one feature to prevent redundancy. This was complemented by a recursive feature elimination strategy, where the most redundant feature was systematically excluded in each iteration.

Univariable Cox Regression: To refine the extensive feature set, we conducted univariable Cox regression analysis. Features were ranked based on their p-values. This method proved more efficacious in enhancing predictive performance than merely selecting features with p-values below 0.05, as demonstrated in our experiments.

Lasso-Cox Regression: In this study, LASSO-Cox regression was used to perform cross-validation on the training set, and the optimal regularization parameter λ was selected through 10-fold cross-validation

Supplementary Figure 1. Gradient-weighted Class Activation Mapping
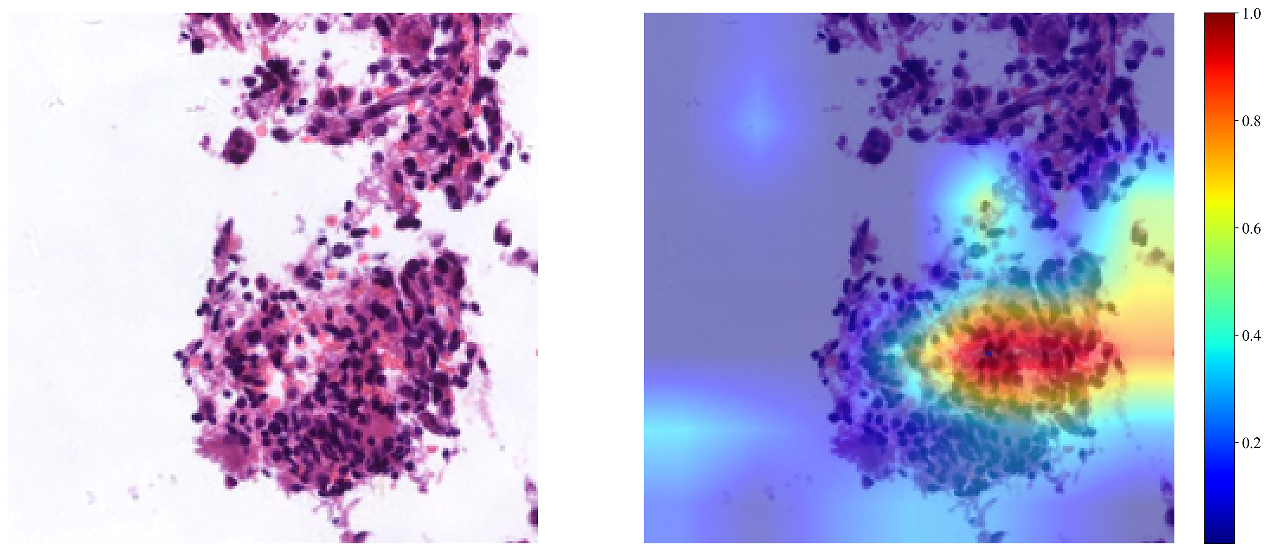


Supplementary Figure 1. Grad-CAM of patch

Supplementary Figure 2. Visualize predictions

This prediction result of prediction 25, 27 sample. It is evident that our pathological model exhibits a high level of accuracy in predicting [TaskSpec] tiles.


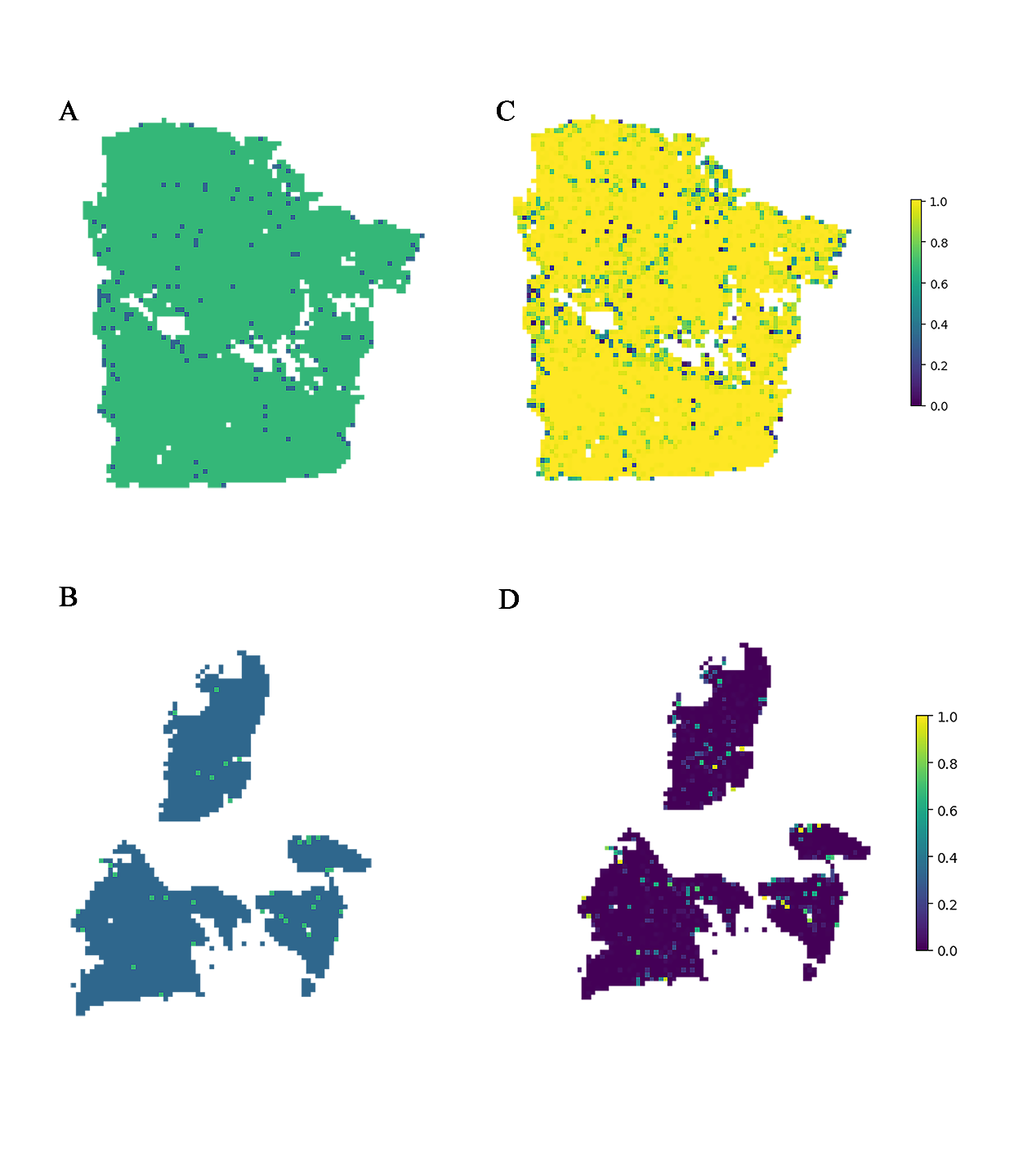


Supplementary Figure 2. Prediction (A, B) and probably (C, D) map of 25(A, C), 27(B, D) sample.

Supplementary Figure 3. Risk stratification and IDH status distinguish Prognosis


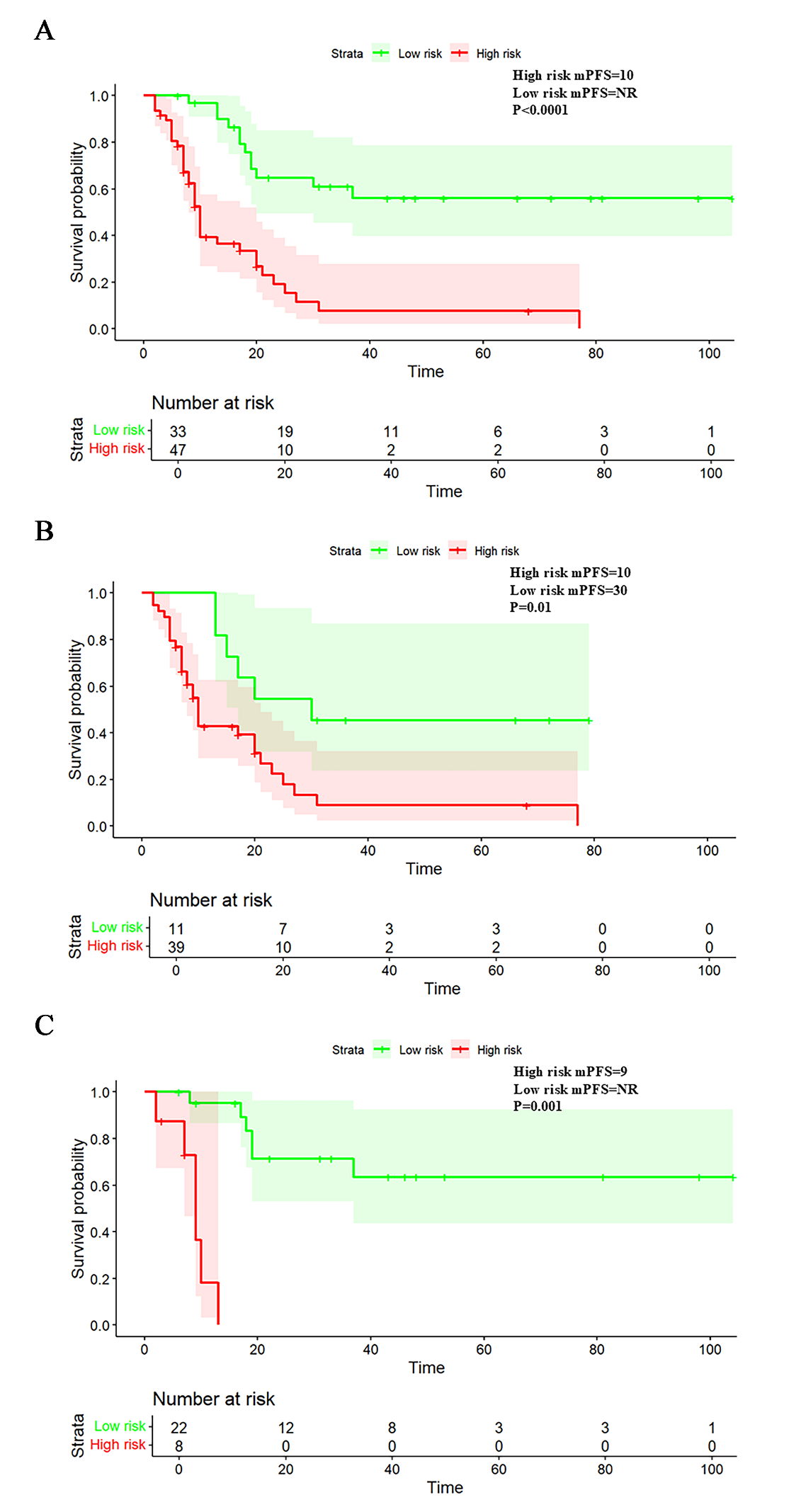


Supplementary Figure 3: (A)Progression Free Survival curve of all patients, (B)Progression Free Survival curve of patients with IDH (-), (C)Progression Free Survival curve of patients with IDH (+). (NOTE:NR:Not Reach)
